# Over or Under? Mental Representations and the Paradox of Body Size Estimation

**DOI:** 10.3389/fpsyg.2021.706313

**Published:** 2021-08-03

**Authors:** Kevin R. Brooks, Richard J. Stevenson, Ian D. Stephen

**Affiliations:** ^1^Body Image & Ingestion Group (BIIG), Faculty of Medicine, Health & Human Sciences, School of Psychological Sciences, Macquarie University, Sydney, NSW, Australia; ^2^Perception in Action Research Centre (PARC), Faculty of Medicine, Health & Human Sciences, Macquarie University, Sydney, NSW, Australia

**Keywords:** adaptation, eating disorders, body size estimation, body size and shape misperception, body representation, aftereffects, body image

## Introduction: the Paradox of Body Size Estimation

In an early body size estimation study, Garner et al. (1976) asked participants with anorexia nervosa to adjust the width of an on-screen photo of their own body until it appeared to match their normal size. When participants chose *larger*-than-veridical bodies as matches, Garner et al. described this as a perceptual overestimation of body size. Forty years later, in an attempt to provide an experimental model of these perceptual experiences, Brooks et al. (2016) exposed participants to photos of thin bodies, simulating the media's portrayal of “thin ideal” figures that have often been blamed for body image distortion (Bruch, 1978). Subsequently, subjects judged the perceived size of photos of themselves that had been manipulated to look larger or smaller. Although subjects judged *smaller*-than-veridical bodies to be the most accurate, this was also described as an overestimation of body size. These studies have been replicated many times, with researchers falling into two camps. For evidence of perceptual overestimation, those with a background in clinical psychology point to the selection of larger matching bodies (Slade, 1985; Cash and Deagle, 1997; Farrell et al., 2005; Gardner and Brown, 2014; Mölbert et al., 2017), while experts in perception offer examples of smaller matching bodies (Winkler and Rhodes, 2005; Glauert et al., 2009; Hummel et al., 2012a,b; Mohr et al., 2016; Stephen et al., 2016, 2018; Ambroziak et al., 2019; Bould et al., 2020; Zopf et al., 2021). How can these opposite patterns of results be described using the same terminology? This apparent paradox can be explained by considering the traditions of the different sub-disciplines of psychology: their assumptions, definitions, and the details of the underlying models of body perception that they employ.

## Conceptual Distinctions and Models of Body Representation

**Figure 1:**
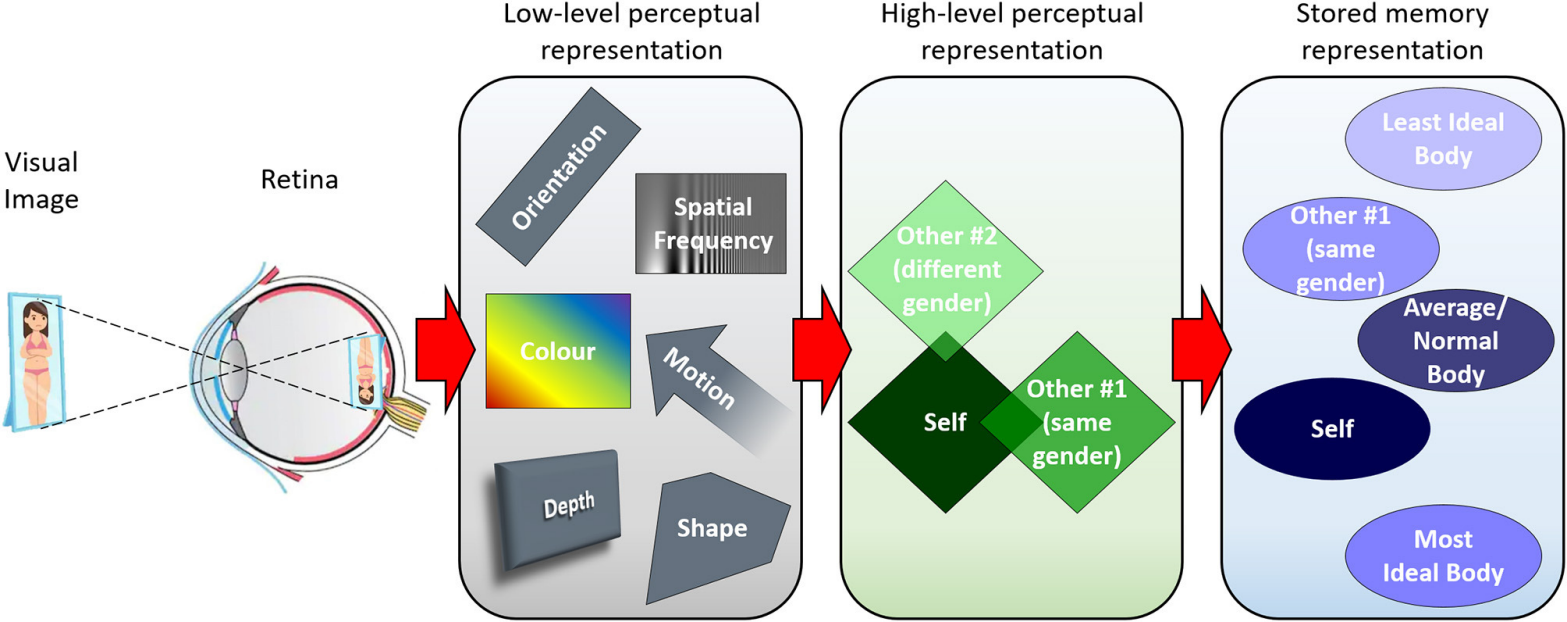
A simple model of visual representations for bodies. Light from objects in the real world forms an image on the retina, where it is transduced into neural impulses. While the stimulus is present, fundamental visual properties are encoded (gray box) before the information is passed on to higher levels of processing where more complex properties are encoded (green box). Bodies perceived thus may then be stored in memory (blue box) for retrieval after removal of the visual stimulus.

Clinical psychologists have long referred to the concept of “body image.” This multidimensional construct includes a perceptual aspect often understood as “the picture of our own body which we form in our mind” (Schilder, 1935/1950). Although the word “perceptual” is often used, in this case body image refers to a representation that is not truly perceptual in the sense in which perceptual psychologists use this word. In the tradition of vision science, the process of forming mental representations progresses as shown in Figure 1. Light from real-world objects (in this case the observer's own body seen in a mirror) forms an image on the retina, where it is transduced into neural impulses. While the stimulus is present, fundamental visual properties are encoded (gray box) before the information progresses to higher processing areas, some of which are body selective. Here, neural activity corresponds to the current perception of more complex properties such as gender, identity, adiposity, muscularity, etc. (green box). Bodies perceived thus may then be stored in memory (blue box) for retrieval when the stimulus is no longer visible. More abstract representations, such as an average or “ideal” body, may be present alongside those that have been encountered explicitly. These may be composites of previously seen bodies [e.g., an average body formed through ensemble coding: Whitney and Yamanashi Leib (2018), Hsieh et al. (2020)], or could be generated spontaneously by one's imagination. In this framework, body image as conceived by Schilder (and many researchers since) corresponds to a stored representation, as it does not require the presence of a visual stimulus, unlike truly perceptual representations.

Consistent with their definition of body image, clinical psychologists' body size estimation tasks often ask participants to manipulate the size of a body (sometimes their own) displayed on a screen to match what they think they currently look like, without observing their own body. Participants make matches between the stored representation of their body and their perception of the body shown on screen, with the unspoken assumption that the latter is veridical. When participants select relatively large on-screen bodies, researchers conclude that the stored representation of their body is larger than their actual size—an overestimation.

Perception researchers take a different approach. This is particularly apparent when the method of adaptation is used. Adaptation involves prolonged exposure to a particular stimulus (known as the adaptor), which then causes an aftereffect of biased perception in a “test stimulus,” whose perceptual properties appear repulsed away from those of the adaptor (Thompson and Burr, 2009). One such example is the well-known motion after-effect, or “waterfall illusion” (Addams, 1834). Viewing of motion in a consistent direction (e.g., the downward motion of a waterfall) for a sustained period (known as adaptation) causes subsequently viewed stationary objects (e.g., nearby rocks) to appear to move upwards. While many adaptation effects concern other basic stimulus properties such as color (Helmholtz, 1924) or line orientation (Gibson and Radner, 1937), aftereffects also apply to more complex stimulus attributes such as the configuration of facial features (Gwinn and Brooks, 2013, 2015a,b), or the adiposity or muscularity of human bodies (Sturman et al., 2017; Brooks et al., 2020a). In psychophysical studies of adaptation, experimenters assume that the perceptual representation of the currently viewed stimulus becomes biased and that the stored representation against which it is implicitly being compared (e.g., stationary rocks, an average face, or a stored representation of one's own body) is veridical. Note that these assumptions are opposite to those made by clinical psychologists. As such, when participants who have been adapted to thin ideal bodies choose a smaller body than their actual size, researchers conclude that the adaptor has caused an aftereffect of expansion for the perceptual representation of the currently viewed on-screen body. This leads the participant to reduce the size of the stimulus to match the stored representation of their body. Although the choice of a smaller on-screen body is opposite to the result observed in clinical body size estimation studies, it is also interpreted as an example of size overestimation.

## Recent Research

Recently (Ambroziak et al., 2019), attempted to explicitly examine the central assumption of body adaptation studies—that adaptation affects the perceptual representation of the stimulus currently being viewed (the test stimulus), not the stored representation of one's own body (the body image). As evidence, they demonstrated that adaptation not only affects body size comparisons between an on-screen test stimulus and the stored representation of one's own body, but also affects comparisons between test stimuli and mental representations of other bodies (e.g., an average body, the experimenter, and Kate Middleton). Aftereffect magnitudes for comparisons with “self” and comparisons with “other” were not significantly different, with Bayesian analyses providing moderate support for the null hypothesis in 4 independent tests. Although similar effects for each condition are consistent with the proposal that adaptation affects the stored representation of all bodies (including one's own, those recently viewed in person, those seen on TV and a composite representation of the average body), the interpretation that adaptation had only affected the perception of the test stimuli on screen is perhaps more parsimonious. However, another aspect of Ambroziak et al.'s results (and of all body adaptation studies mentioned so far) argues even more persuasively for an effect of adaptation on test stimuli rather than internal representations—the direction of the change in perceived size, as explained above. The test stimulus perceived to match the stored reference body was objectively *smaller* after exposure to thin adaptors—an observation that can only be explained as an aftereffect of repulsion on the test stimuli. A repulsive aftereffect on the stored body image would result in the opposite pattern of results—an *increase* in the size of the body perceived to match the stored reference body.

Other data that are consistent with the interpretation that body adaptation affects the perception of currently viewed test stimuli rather than the stored representation of one's own body have recently been obtained in our laboratory (Zopf et al., 2021). This study sought evidence of cross-modal transfer of the visual body size aftereffect to the tactile domain. If adaptation affects the stored representation, it is conceivable that the effect could transfer to judgements of the distance between two tactile stimuli applied to the abdomen. While adaptation to large and small bodies produced the expected repulsive size aftereffects on test images depicting the participant's own body, this did not transfer to tactile distance estimates. This result is again consistent with the idea that body adaptation affects the perception of test stimuli, not the body image. While one could argue that adaptation may affect a stored representation of the body that is unimodal (purely visual), again the direction of the effect makes this suggestion untenable.

## Adaptation in Real-World Cases of Body Image Distortion

Several authors have suggested that adaptation may serve as a laboratory model of, a treatment for, or even the cause of real-world examples of body image distortion (Winkler and Rhodes, 2005; Glauert et al., 2009; Hummel et al., 2012b; Brooks et al., 2016; Mohr et al., 2016; Challinor et al., 2017; Stephen et al., 2018; Bould et al., 2020). However, the discrepancy between results in adaptation studies and those in clinical studies may appear to cast doubt on these claims. Nevertheless, it remains possible that adaptation could play a role in examples of body image distortion if the size aftereffect were transferred from the perceptual representation to the stored representation, as suggested by Brooks et al. (2020b). For example, if an individual perused “thinspiration” images on social media before observing him/herself in a mirror or photograph, they would be likely to perceive their body to be larger than it is. This enlarged percept may then be used to update the stored representation of their own body, and the overestimation of body size would be fixed in memory. If this individual had been asked to adjust the mirror image (serving as the test image) to match their original perceived size, they would have reduced its size. Were they to adjust a “test” body to match their stored representation after the adaptation had subsided, they would need to embiggen it. This example illustrates how the two different patterns of “overestimation” results can coexist, while presenting a mechanism through which adaptation might produce situations where the stored body representation becomes distorted in a way that corresponds with observations in many clinical body size estimation studies.

## Discussion

In this article, we have introduced the paradox of body size estimation—that two opposite patterns of results have been described as overestimation by different groups of scientists—and explained this in terms of two distinct body representations: a perceptual representation and a stored memory representation. Finally, we have described a situation in which an adaptation-induced distortion in the former representation may be transferred to the latter, thus providing a means by which adaptation may apply to real-world examples of body image distortion. Whether or not adaptation is relevant to cases such as these has yet to be determined, but even if it is not, this technique remains an invaluable non-invasive method of probing the brain mechanisms underlying body perception. So far, adaptation studies have revealed these mechanisms to be high level (Hummel et al., 2012b; Brooks et al., 2018), and selective for identity (Brooks et al., 2016) and gender (Brooks et al., 2019, 2020a), yet they generalize across race (Gould-Fensom et al., 2019). There also appear to be independent neural populations responsible for the perception of fat and muscle mass (Sturman et al., 2017; Brooks et al., 2020a). Further, these mechanisms are moderated by attention (Stephen et al., 2018, 2019). Moreover, we believe that improved general awareness of the effects of adaptation would benefit many body image researchers. In designing and analyzing the results of experiments on media saturated with specific body types (size zero models, muscular figures, etc.) it is essential to realize that exposure to these idealized bodies is likely to affect what participants see, regardless of whether it affects the stored body image *per se*.

## Author Contributions

KB responsible for conception, original drafting, creation of the figure, and final approval of the submitted version. IS and RS responsible for contribution of original ideas and final approval of the submitted version.

## Conflict of Interest

The authors declare that the research was conducted in the absence of any commercial or financial relationships that could be construed as a potential conflict of interest.

## Publisher's Note

All claims expressed in this article are solely those of the authors and do not necessarily represent those of their affiliated organizations, or those of the publisher, the editors and the reviewers. Any product that may be evaluated in this article, or claim that may be made by its manufacturer, is not guaranteed or endorsed by the publisher.
